# Loss of Function of *OsFBX267* and *OsGA20ox2* in Rice Promotes Early Maturing and Semi-Dwarfism in γ-Irradiated IWP and Genome-Edited Pusa Basmati-1

**DOI:** 10.3389/fpls.2021.714066

**Published:** 2021-09-22

**Authors:** M. T. Andrew-Peter-Leon, Ramchander Selvaraj, K. K. Kumar, Mehanathan Muthamilarasan, Jeshima Khan Yasin, M. Arumugam Pillai

**Affiliations:** ^1^Department of Plant Breeding and Genetics, Agricultural College and Research Institute, Tamil Nadu Agricultural University, Tuticorin, India; ^2^Centre for Plant Molecular Biology and Biotechnology, Tamil Nadu Agricultural University, Coimbatore, India; ^3^Department of Plant Sciences, School of Life Sciences, University of Hyderabad, Hyderabad, India; ^4^Division of Genomic Resources, ICAR-Indian Council of Agricultural Research, National Bureau of Plant Genetic Resources, New Delhi, India

**Keywords:** dwarfing, early flowering, F-box protein, genome editing, genome sequencing, *Sd1*

## Abstract

Targeted mutagenesis is now becoming the most favored methodology to improve traits in popular rice cultivars selectively. Understanding the genetic basis of already available mutants could be the first step in designing such experiment. Improved White Ponni (IWP), a popularly grown South Indian rice variety, was subjected to γ irradiation to develop WP-22-2, an M_6_ line superior in semi-dwarfism, early flowering, and high yield, and it has grain qualities similar to those of IWP. The exogenous application of gibberellic acid (GA_3_) on WP-22-2 resulted in the elongation of shorter internodes to a level similar to IWP. The expression profiling of six genes regulating plant height showed their differential expression pattern at different time points post GA_3_ treatment. Furthermore, the sequencing of WP-22-2 and IWP genomes revealed several single nucleotide polymorphisms (SNPs) and large-scale deletions in WP-22-2. The conversion of functional codons to stop codons was observed in *OsGA20ox2* and *OsFBX267*, which have been reported to have roles in regulating semi-dwarfism and early flowering, respectively. The loss of function of *OsGA20ox2* and *OsFBX267* in WP-22-2 resulted in reduced plant height as well as early flowering, and the same has been confirmed by editing *OsGA20ox2* in the rice variety Pusa Basmati1 (PB1) using the CRISPR-Cas9 approach. The targeted editing of *OsGA20ox2* in PB1 conferred shorter plant height to the edited lines compared with the wild type. Altogether, the study provides evidence on mutating *OsGA20ox2* and *OsFBX267* genes to develop early maturing and semi-dwarf varieties that can be released to farmers after functional characterization and field trials.

## Introduction

Improved White Ponni is a medium-duration rice variety widely cultivated in Southern India. The high commercial consumer preference of this variety is due to superior grain quality traits, such as fine grain structure and high head rice recovery. This variety also has high yield potential and resistance to diseases such as rice tungro bacilliform virus infection, leaf yellowing, blast, and bacterial leaf blight (Rajendran, 2014). As Improved White Ponni (IWP) is tall (>140 cm in height) and has a medium duration in maturity, the variety is prone to lodging, which causes heavy yield loss to farmers (Subramanian et al., 1986). Thus, improving the architecture of the plant by reducing plant height and days-to-maturity could significantly enhance lodging resistance and prevent yield loss. Among the different approaches deployed for trait improvement, mutation breeding has been a preferred and successful method. Gamma-ray-induced mutations were shown to be effective in altering the improvement of agronomic growth and stress tolerance traits of several crops, which include rice. Such mutant lines were released as new varieties for commercial cultivation or had been used as donors of improved traits in breeding programs (Ahloowalia et al., 2004). Although the phenotypic characteristics of the gamma-induced mutants are extensively studied, the nucleotide-level changes hold the key to understanding the alterations in the genes that confer those specific phenotypes.

Gamma-ray irradiation causes genome-wide mutations, wherein identifying genes that underwent base changes is imperative to understand the molecular roles of those genes. Although conventional marker systems, such as simple-sequence repeats (SSRs), could identify major mutation sites, the resolution of those markers is not powerful enough to identify minor variations and single-base changes in the genes. This bottleneck was substantially ameliorated by the intervention of next-generation sequencing (NGS), as it allowed for the identification of genome-wide variations at base pair-level in the mutants (Barabaschi et al., 2016; Heuermann et al., 2019). Being the first crop genome to be sequenced, rice has gold-standard sequence data and enormous sequence information available in public domains (Eckardt, 2000; IRGSP, 2005; Song et al., 2018). This is a valuable resource for deploying comparative genomics approaches to compare any mutant genotype with the wild type to identify the mutations underlying the genetic determinants of improved traits in the mutants. Further characterization of those mutant genes derives their precise roles in regulating the desired phenotype, further aiding in breeding programs.

Dwarf plant type and short duration are two important attributes that breeding programs have been targeting to achieve in rice. IR8, a semi-dwarf cultivar released by the International Rice Research Institute in the Philippines has become the most popular cultivar in research to study these traits. The incomplete recessive gene, *d47*, caused semi-dwarfism in Dee-Geo-Woo-Gen, the parent line of IR8. Later, the recessive gene in the Calrose 76 variety was found to be *sd1*, which was allelic to *d47*. Similar allelic relationships were found in cv. Taichung Native 1 derived from Dee-Geo-Woo-Gen and cv. Shiranui derived from Jukkoku. The *d49* identified in the mutant cultivar Reimei was also allelic to *sd1*. It was proven that *sd1* is the most common locus that controls semi-dwarfism despite having different parentage (Tomita and Ishii, 2018). Despite this information, several other genes in the gibberellin pathway or that epistatically control the pathway have been known to control plant height in rice (Aach et al., 1997; Hedden and Phillips, 2000; Helliwell et al., 2001; Hong et al., 2005; Ishikawa et al., 2005; Ueguchi-Tanaka et al., 2005; Yamaguchi, 2008; Hedden and Sponsel, 2015). In the case of flowering, several studies have reported the genetic control of flowering time in rice. The FLOWERING LOCUS T/Heading date3a (*Hd3a*) protein identified in rice and *Arabidopsis* constitutes the flowering signal called florigen (Turck et al., 2008). In rice, *Hd3a* has functional variations because of its facultative short-day nature, and it is activated by several unique genes that are non-orthologous to *Arabidopsis*, such as *Ehd1, Ehd2, Ehd3, Ehd4, Ghd7*, and *MADS51*. These genes mediate the florigen pathway and control flowering time (Doi et al., 2004). Further, regulatory genes such as *OsLFL1* (Ryu et al., 2009), *OsMADS14* (Kyozuka et al., 2000), and *OsFKF1* (Han et al., 2015) are known to control flowering time in rice. However, because of the complex mechanisms regulating this trait, comprehensive genome-wide studies are essential to gain mechanistic insights into flowering and early maturity.

The previous study showed that the WP-22-2 mutant is sensitive to gibberellic acid (Andrew-Peter-Leon et al., 2021). Responsiveness to exogenous gibberellin (as measured by the reversal of internode elongation in dwarf mutants) indicates a defective gibberellin pathway in rice (Ashikari et al., 2002), and the famous mutant *sd1*allele is also sensitive to gibberellin. This study aimed to validate these observations further.

In this study, stable mutant lines of IWP with dwarf plant type and short duration were developed with γ-irradiation. The sequencing of mutant and wild-type lines detected mutations in the *OsFBX267* and *OsGA20ox2* genes subjected to further characterization. The results showed that the two genes regulate dwarf plant type and earliness in flowering, and further editing of *OsGA20ox2* using CRISPR-Cas9 in Pusa Basmati 1 validated the experimental findings of this study. Altogether, this study has identified two players that regulate dwarf plant type and earliness in flowering in rice, which could be further exploited in crop improvement programmes.

## Materials and Methods

### Genetic Material

This study used two rice cultivars popularly cultivated in South (Improved White Ponni) and North India (Pusa Basmati 1) as experimental materials. IWP is a medium-duration (115 days to flowering) cultivar with fine slender grains (Subramanian et al., 1986). The seed materials were collected from the germplasm collections of the Tamil Nadu Agricultural University. The plants were raised at the Agricultural College and Research Institute, Killikulam, and Agricultural Research Station, Thirupathisaram, from 2011 to 2016.

### Mutagenesis, Selection, and Evaluation of Mutant Lines

Five hundred well-filled seeds of IWP were treated with different doses of gamma rays (100, 200, 300, 400, and 500 Gy) using Gamma Chamber (Model GC 1200, BRIT, India) installed at the Tamil Nadu Agricultural University, Coimbatore, India. Immediately after the treatment, the seeds were allowed to germinate in the fields of Agricultural College and Research Institute, Killikulam, India, following standard agricultural practices. Non-mutagenized IWP wild-type plants were also raised as control. The M1 plants were harvested individually at maturity, and 184 plants were advanced to M_2_ in 2012. In M_2_, 152 early flowering and dwarf mutants were identified and advanced to M_3_ generation for further evaluation and validation. The M_3_ population was evaluated for a reduction in days to flowering (early flowering), dwarfism, high yield, and fine grain quality traits compared with the IWP. In the M_5_th generation, 70 mutants were chosen and screened for high yield and quality traits similar to IWP. Twenty such mutants were forwarded to the M_6_ generation. In M_6_ generation, the mutants were evaluated for morphological characteristics, such as plant height (PH, in cm), days to 50% flowering (DFF), number of productive tillers (NOPT), panicle length (PL, in cm), number of grains per panicle (GPP), single plant yield (SPY, in g), and 1,000-grain weight (TGW, in g). Furthermore, grain quality traits, namely, milling percent, head rice recovery (HRR) percent, grain length before cooking (LBC, in mm), grain breadth before cooking (BBC, in mm), grain length and breadth ratio (LB), grain length after cooking (LAC, in mm), grain breadth after cooking (BBC, in mm), linear elongation ratio (LER), breadthwise elongation ratio (BER), and alkali spreading value (ASV) were also evaluated. The chlorophyll content of the genotypes were measured (in SPAD units) using a portable chlorophyll meter (Soil Plant Analytical Development, SPAD, Model 502). The average leaf area (in cm^2^) was measured by following the standard method (Yoshida, 1981).

As all the selected mutants were fixed and found to be stable in M_6_ generation, a mutant designated as WP-22-2 was chosen for multiplication for further analyses. WP-22-2 was found to be superior in semi-dwarfism, early flowering, high yield, and similar grain qualities of IWP (fine-slender grains and cooking quality).

### Exogenous Application of Gibberellic Acid and Expression Profiling by qRT-PCR

Earlier, we reported the high sensitivity of WP-22-2 to 50 μM gibberellic acid (Andrew-Peter-Leon et al., 2021). This was tested by raising WP-22-2 along with IWP (wild-type) in a completely randomized design and spraying the 10-day-old seedlings with 50 μM of GA_3_. Untreated plants served as the control, and three biological replicates for five technical replicates were maintained. Five days post sowing, the seedling height in cm, first internode length (in cm), and second leaf length (in cm) were measured and analyzed by Student's *t*-test.

For expression profiling, the leaves were harvested from treated and control plants 0, 6, 12, and 24 h post spraying of GA_3_, and total RNA was extracted following standard procedures (Chomczynski and Mackey, 1995). Following the instructions of the manufacturer, the first-strand cDNA synthesis was performed using the Protoscript M-MuLVRT kit (NEB, Ipswich, MA, United States). Gene-specific primers for six regulatory genes involved in the gibberellin pathway of rice (*OsSLR1*, LOC_Os03g49990; *OsGA20Ox2*, LOC_Os01g66100; *OsKOL4*, LOC_Os06g37300; *OsKO2*, LOC_Os06g37364; *OsMAX2*, LOC_Os06g06050; *OsBRD2*, LOC_Os10g24780) were developed using GenScript Real-time PCR Primer Design tool (https://www.genscript.com/ssl-bin/app/primer) with default parameters. The qRT-PCR analysis was performed using SYBR Green detection chemistry on 7900HT Sequence Detection System (Applied Biosystems, Waltham, MA, United States). The reaction mixture in a final volume of 20 μl containing 2 μl of 5X diluted cDNA, 250 nM of each primer, and 10 μl of Power SYBR Green PCR Master Mix (Applied Biosystems, Waltham, MA, United States) was subjected to initial denaturation for 2 min at 50°C and 10 min at 95°C, and 40 cycles of 15 s at 95°C and 1 min at 60°C. Three technical replicates for three independent biological replicates were maintained during the experiment.

### Whole-Genome Sequencing and Processing of Sequence Data

Total DNA was isolated from IWP and WP-22-2 following the standard CTAB method, and the quality and quantity of the samples were ascertained by resolving on 1.2% agarose gel and with a NanoDrop1000 spectrophotometer (Thermo Fisher Scientific, Waltham, MA, United States), respectively. The DNA samples were then subjected to sequencing at 30X coverage on the Illumina HiSeq2500 platform (AgriGenome Labs Pvt Ltd, Hyderabad, India). Based on previous reports, more than 100 potential sites that control plant height and days to maturity in rice were chosen; their promoter and gene sequences were compared against the IWP and WP-22-2 and analyzed for indels and SNPs. SNPs that qualify in SNP calling were tested for their significance and filtered to obtain high-quality SNPs. Mutations identified in three genes, namely, *B3DNA* (LOC_Os03g42290), *OsFBX267* (LOC_Os08g09466), and *OsGA20ox2* (LOC_Os01g66100), were found to have frameshifts or premature terminations that affected the protein synthesis. These were further confirmed by Sanger sequencing. A multiple sequence alignment of Sanger sequencing data and corresponding reference genes was performed using MUSCLE (Madeira et al., 2019) under default parameters. The data was further analyzed on BioEdit (Hall, 1999). The genes and sequence variations were illustrated using Illustrator for Biological Sequences (Liu et al., 2015).

### Annotation and Analysis of Candidate Genes and Proteins

The gene sequences were BLAST-searched against rice genome (release 7) available in the Rice Genome Annotation Project database (http://rice.plantbiology.msu.edu/analyses_search_blast.shtml) to identify the CDS (coding DNA sequence) of each gene. Furthermore, the alignment of candidate genes sequenced from IWP and WP-22-2 was performed in the LALIGN server (https://embnet.vital-it.ch/software/LALIGN_form.html). The DNA sequences were then translated to amino acid sequences using EMBOSS Transeq (https://www.ebi.ac.uk/Tools/st/emboss_transeq/) and EMBOSS Sixpack (https://www.ebi.ac.uk/Tools/st/emboss_sixpack/) (Madeira et al., 2019). The predicted protein sequences were aligned using EMBOSS Needle (https://www.ebi.ac.uk/Tools/psa/emboss_needle/). The SNPs identified were classified as synonymous (coding for the same amino acid) and non-synonymous (coding for a different amino acid) based on amino acid changes. The substituted SNPs were analyzed for their functional effects using Sorting Intolerant From Tolerant (SIFT: https://sift.bii.a-star.edu.sg/www/SIFT_seq_submit2.html) (Ng and Henikoff, 2003) and sorted as “tolerated” (score:.05 to 1) or “deleterious” (score: 0–0.05) according to their scores. The SIFT prediction was compared with PROVEAN (http://provean.jcvi.org/seq_submit.php) for similar functional effects (Choi et al., 2012; Choi and Chan, 2015) with a threshold value of −2.5, below which a particular mutation was considered deleterious. Furthermore, protein 3D-structure was predicted using Phyre2 (http://www.sbg.bio.ic.ac.uk/phyre2/) (Kelley et al., 2015).

### Targeted Editing of *OsGA20ox2* in Pusa Basmati

*OsGA20ox2* (LOC_Os01g66100), popularly known as “Semi-dwarf 1”, has three exons in its coding region. Mutations (SNPs and indels) in this gene cause dwarfism in rice cultivars (Sasaki et al., 2002). The first exon of this gene was targeted for gene silencing in the rice cultivar Pusa Basmati-1 (PB-1). The CRISPR-Cas9 vector, pRGEB32, from Yinong Yang (Xie et al., 2015) was acquired through Addgene (Watertown, MA, United States) (Addgene plasmid #63142; http://n2t.net/addgene:63142; RRID: Addgene_63142). A unique 20-nucleotide guide sequence for the targeted region was designed using the CRISPR-PLANT tool (http://www.genome.arizona.edu/crispr) and following the standard procedures (Ren et al., 2014; Doench, 2017). The target regions were selected to have suitable restriction enzyme sites at the Cas9 endonuclease cutting site (3-bp upstream 5′-NGG) to detect genome editing in the plants by PCR/RE assay (**Figure 6**). The constructs were prepared following the standard protocol (Xie et al., 2014), and then transformed into an *Agrobacterium tumefaciens* AGL-1 strain using the triparental mating method (Ditta et al., 1980). Well-matured and healthy seeds of PB-1 were cultured to produce transformable calli on a Murashige and Skoog medium (Murashige and Skoog, 1962), with minor modifications. An *Agrobacterium-*mediated genetic transformation was performed following an established protocol (Kumar et al., 2005). The transformants were screened for the presence of hygromycin phosphotransferase gene (*hptII*) by PCR, and the mutants were characterized using gene-specific PCR primers (Supplementary Material 13). The genome-edited lines as well as the control plants (three technical replicates for three independent biological replicates) were further phenotyped for PH and other parameters.

### Statistical Analysis

#### Biometrical Data

The biometrical data were obtained from replicated trials (randomized block designs), and the variability parameters, such as phenotypic and genotypic coefficient of variation, heritability, and genetic advance, were calculated following standard methods (Lush, 1940; Burton, 1952; Johnson et al., 1955; Panse et al., 1961).

The mean data of IWP and WP-22-2 in different experiments were compared by Students *t-*test for significant differences in the R statistical program [(R Core-Team, 2018) and ggplot2 (Wickham, 2016)].

#### Quantitative Real-Time PCR

The PCR efficiency was calculated with the default software itself (Applied Biosystems, Waltham, MA, United States). The transcript abundance of the qRT PCR, normalized to the endogenous control *OsActin*, was analyzed using the 2^−Δ*ΔCt*^ method (Livak and Schmittgen, 2001).

#### Whole-Genome Sequencing Data

The raw reads were processed for adaptor removal and quality trimming using AdapterRemoval (version 2.2.0). A paired-end alignment of high-quality reads to the reference genome (“Nipponbare”v7.0; http://rice.plantbiology.msu.edu/) was performed using Bowtie2 (version 2-2.2.9) with default parameters, and variant calling was performed with default settings of SAMtools (version 0.1.18).

## Results

In this study, IWP mutants were developed as explained in the previous reports (Ramchander et al., 2014, 2015a,b), and the mutant lines were phenotyped for several yield-contributing agronomic traits with emphasis on plant height and short duration. Significant variations in these traits were observed in M_2_ and M_3_ generations. Mutants exposed to 100 Gy of γ irradiation recorded the lowest 50% flowering (106.2 mean days), while 300 Gy recorded the lowest plant height of 138.7 cm (Supplementary Material 1). M_3_ of 100, 200, and 300 Gy irradiation showed high heritability and genetic advance for the traits, namely the number of grains per panicle, primary culm length, secondary culm length, and first, second, third, and fourth internode lengths (Supplementary Materials 2, 3).

### Identification of Mutant Lines for Desired Phenotypes

**Figure 1:**
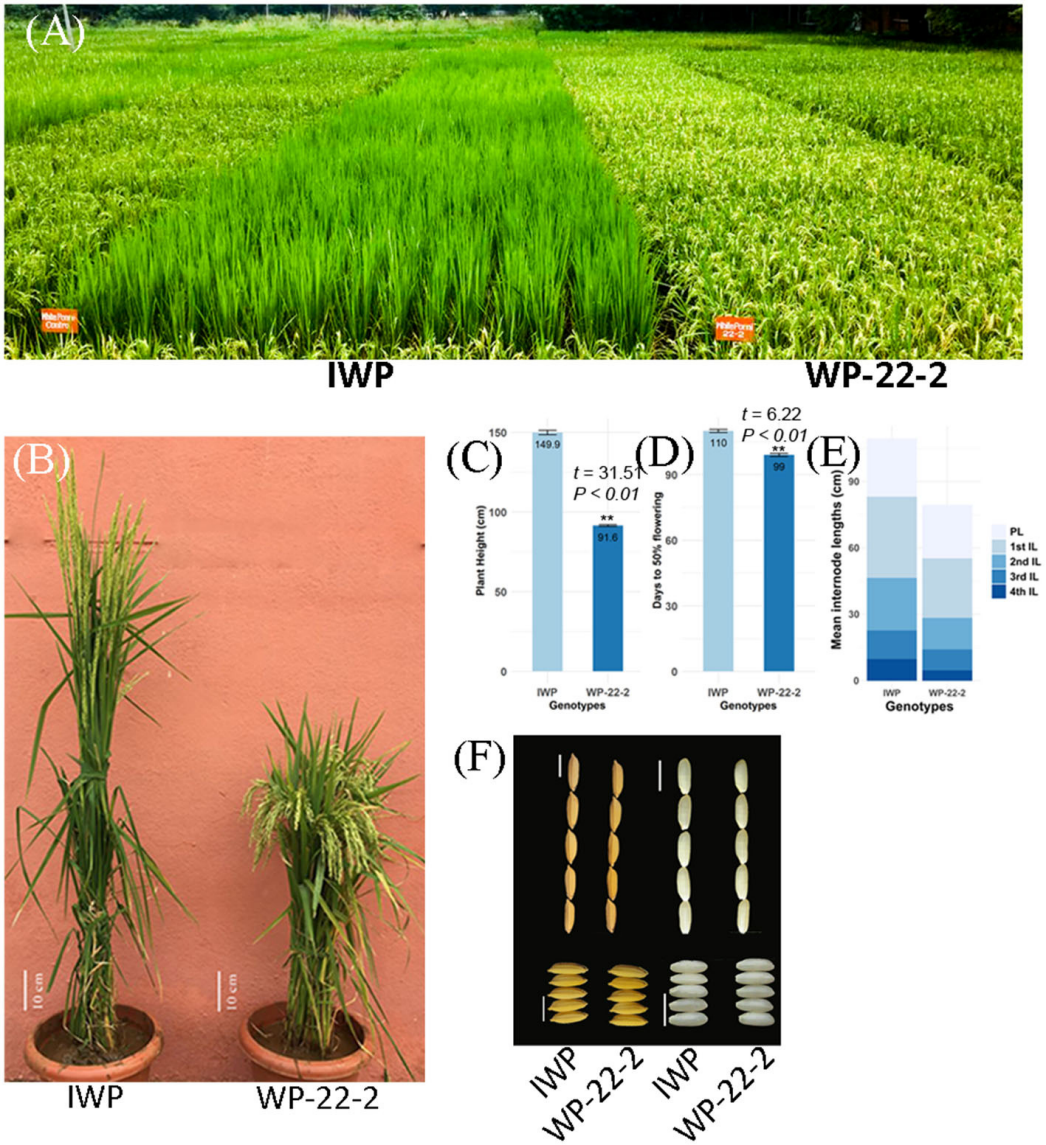
Comparison between the rice variety Improved White Ponni (IWP) and superior mutant WP-22-2. **(A)** Field view of IWP and WP-22-2. IWP is clearly tall and still in vegetative stage, whereas WP-22-2 is dwarf and has already reached maturity. **(B)** Plants of IWP and WP-22-2 (scale bar: 10 cm). **(C,D)** Barplot showing difference in days to 50% flowering and plant height between c- IWP and m-WP-22-2. **(E)** Comparison of internode and panicle lengths of IWP and WP-22-2. **(F)** Length and breadth of rice grains of IWP and WP-22-2 (scale bars: 5 mm).

Several mutants recorded lesser duration of flowering (ranging from 79 to 92 days), dwarf plants (85–105 cm plant height), and better tillering ability (32–44 tillers) with fine grains (Supplementary Material 3). The lengths of the first four internodes of the mutants showed significant variations. The first internode length of the mutants ranged from 16.6 to 42 cm, while it was 35.3 cm in IWP-control. The second internode length ranged from 11.5 to 40.1 cm in the mutants. At M_6_ generation, WP-22-2 mutant showed superior phenotypes, including days to flowering (99 days), PH (91.6 cm), and SPY (54.55 g), and cooking quality traits similar to those of the IWP control (Figure 1, Table 1). The IWP control plants showed 110 days to flowering, 149.9 cm PH, and 40.79 g SPY. The average leaf area and the chlorophyll content were also similar in the WP-22-2 mutant and IWP. Thus, considering the agronomic superiority of WP-22-2 over IWP, WP-22-2 was chosen for further characterization to understand the molecular basis of semi-dwarfism and earliness in flowering.

**Table 1:** Comparison of different traits between Improved White Ponni (IWP) and WP-22-2 in the generation of M_6_.

**Traits**	**IWP**	**WP-22-2**
Days to 50% flowering	110	99
Plant height (cm)	149.9	91.6
Number of productive tillers	21.65	21.25
Panicle length (cm)	26.3	24.2
Grains per panicle	224.5	264.25
Single plant yield (g)	40.79	54.55
Thousand grain weight (g)	15.6	12.45
Milling (%)	61.7	66.65
Head rice recovery (%)	53.05	57.6
Grain length (mm)	5.45	4.95
Grain breadth (mm)	2	1.85
LB ratio	2.73	2.68
Grain length after cooking (mm)	8.1	7.2
Grain breadth after cooking (mm)	2.75	2.5
Linear elongation ratio	1.49	1.45
Breadthwise elongation ratio	1.38	1.35
Alkali spreading value	3	3
Chlorophyll content (SPAD units)	32.96	31.97
Average leaf area (cm^2^)	25.64	24.42

### Morphological and Molecular Response of WP-22-2 to Exogenous GA_3_ Treatment

**Figure 2:**
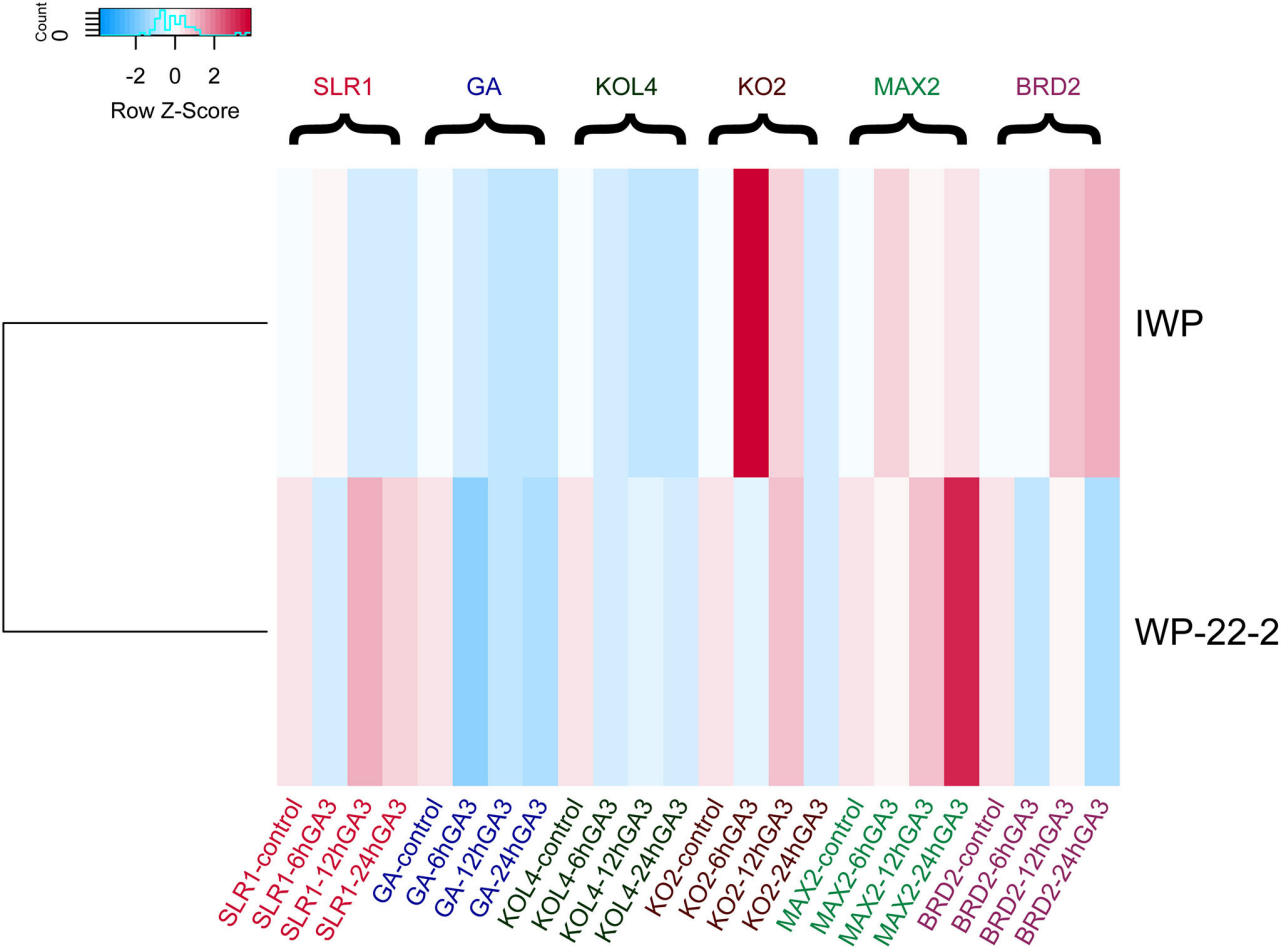
Heatmap of the gene expression data of IWP and WP-22-2. ddC_T_ values of the quantitative real time polymerase chain reaction (qRT-PCR) were plotted. Clear expression changes were observed for the genes KO2 (ent-kaurene oxidase2), MAX2 (Fbox/LRR repeat MAX2 homolog), and brassinosteroid deficient 2 (BRD2) between IWP and the mutant WP-22-2.

In the earlier study, we identified that WP-22-2 was highly sensitive to gibberellin (Supplementary Material 4) compared with IWP (Andrew-Peter-Leon et al., 2021) (Figure 2). Therefore, the effect of the exogenous application of GA_3_ (50 μM) was studied through foliar spray on WP-22-2. Seedlings at the two-leaf stage (15 days old) had shorter internodes (1st and 2nd) than IWP; however, GA_3_ application resulted in leaf elongation at these internodes to a level similar to the wild-type, IWP. To gain further insights into this, the expression of six genes playing roles in regulating plant height was studied in WP-22-2 and IWP treated with GA_3_. Overall, the data showed a differential expression pattern of all the genes examined (Figure 2, Supplementary Materials 5–7). A notable upregulation of *OsSLR* was observed in the WP-22-2 12-h sample compared with IWP. However, there are no noticeable changes in the expression of *OsKOL4* throughout all the time points of WP-22-2 and IWP. The expression levels *OsKOL2* were similar in both WP-22-2 and IWP across the time points except in the 6-h sample of IWP, where a significant upregulation was observed. Similarly, *OsMAX2* was found to be upregulated in the 24-h sample of WP-22-2 compared with IWP. Interestingly, *OsBRD2*, a negative regulator of gibberellin metabolism, showed downregulated expression in WP-22-2 upon GA_3_ application (*P* < 0.01) (Figure 2).

### Low-Throughput Genome Sequencing and Analysis of Sequence Data

**Figure 3:**
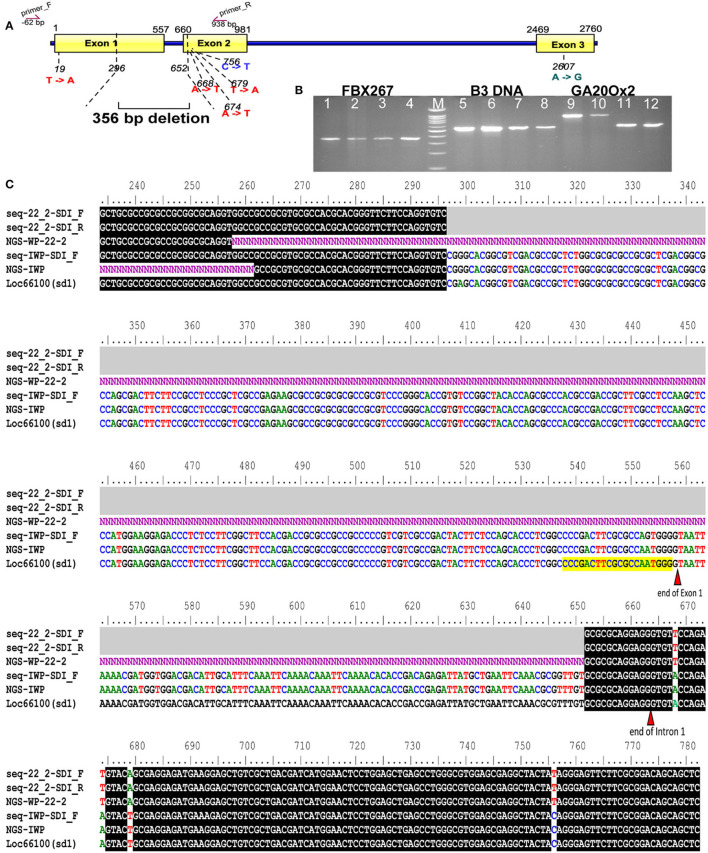
Gene illustration and sequence alignment of SD1 locus. **(A)** Gene illustration showing the regions of deletion and other single nucleotide variations between IWP and WP-22-2; binding sites of the primer used for Sanger sequencing are marked as arrows above the gene diagram. **(B)** Cropped gel image of B3 DNA, FBX267, and GA20Ox2 gene regions of IWP and WP-22-2 (biological duplicates) amplified with PCR primers; IWP: 1 and 2, 5 and 6, 9 and 10; WP-22-2: 3 and 4, 7 and 8, 11 and 12; M: 100 bp ladder—each band represents a 100-bp increment from the lower band; a large deletion is observed between IWP and WP-22-2 in the GA20Ox gene (Full-length gel image is presented in Supplementary Material 10). **(C)** Sequence alignments showing single nucleotide variations. The horizontal gray bars indicate (in seq-22_2-SD1_F and seq-22_2-SD1_R) deletion. *Order of alignment: Sanger (WP-22-2_F&R), NGS WP-22-2, Sanger IWP, NGS IWP and annotated Nipponbare LOC_Os01g66100*.

The genomes of WP-22-2 and IWP were sequenced using the Illumina platform, and the sequence reads were compared to identify SNPs and indels. A total of 38 SNPs and a prominent 356-bp deletion were observed in the coding regions of B3 DNA binding domain (LOC_Os03g42290), *OsFBX267* (LOC_Os08g09460), and *OsGA20ox2* (LOC_Os01g66100) genes. Comparing sequence data of Nipponbare, IWP, and WP-22-2 showed 15 SNPs in the seven exons of the B3 DNA binding domain. However, a single SNP at the seventh exon (G/G/A at position 5231 bp of B3 DNA loci) was unique to WP-22-2 (Table 2, Supplementary Material 8). Similarly, the SNPs observed at 17 sites of the three exons of *OsFBX267* (LOC_Os08g09460) were unique to WP-22-2 (Table 2, Supplementary Material 9). Studying the nature of base change showed that changes in Cs (cytosines) and Ts (Thymines) were predominantly present. In *OsGA20ox2*, a large deletion of 356 bp was observed in WP-22-2, and this deletion included 262 nucleotides at the 3′ end of exon 1, complete intron 1 (103 nucleotides), and an 18-nucleotide deletion at the 5' end of exon 2 starting from the 296th to 652nd bp of the gene. In addition, two SNPs at exon 2 (T/T/A at position 679 and C/C/T at position 756 bp: Nipponbare/IWP/WP-22-2, respectively) were found to be unique to WP-22-2 (Figure 3, Table 3).

**Table 2:** Single nucleotide polymorphisms (SNPs) observed in the B3 DNA and OsFBX267 gene coding regions of Nipponbare, IWP, and WP-22-2 genotypes.

**Gene**	**Position**	**SNPs (Nipponbare/IWP/WP-22-2)**	**Base position in the gene (from the start of the codon “ATG”)**
B3 DNA (LOC_Os03g42290)	Exon 1	G/A/A	20
	Exon 3	G/A/A	2358
		C/T/T	2414
		A/G/G	2532
		A/C/C	2578
		T/C/C	2670
		C/A/A	2719
		A/C/C	2732
	Exon 5	C/T/T	3871
		G/T/G	3828
	Exon 7	T/C/C	4996
		G/T/A	5132
		C/T/T	5173
		G/G/A	5231
		C/A/N	5344
OsFBX267 (LOC_Os08g09460)	Exon 1	T/T/C	36
		C/C/T	41
		G/G/A	44
	Exon 2	T/T/C	457
		G/G/C	464
		C/C/T	475
		T/T/C	489
		C/C/T	516
		T/A/G	523
		A/A/C	535
		C/C/A	543
		C/C/G	622
		C/C/T	624
		C/C/T	636
		C/C/A	677
	Exon 3	G/G/A	1487
		T/T/C	1530
		T/T/C	1556
		T/T/C	1579
		G/G/C	2068

**Table 3:** Details of mutations in the OsGA20ox2 gene of WP-22-2.

**S.No**.	**Gene location**	**Annotated gene characteristics (as per IRGSP 1.0)**	**Sequence variations in IWP**	**Sequence variations in WP-22-2**
	Deletions			
1.	Exon 1	558bp length	Nil	262 bp deletion at the 3′ end
2.	Intron 1	102bp length	Nil	93 bp deletion at the 5′ end
	**Single nucleotide variations**			
1.	Exon 2	'A' at the 5th base of exon 2	'A'	A → T
2.		'A' at the 11th base of exon 2	'A'	A → T
3.		'T' at the 16th base of exon 2	'T'	T → A
4.		'C' at the 93rd base of exon 2	'C'	C → T
5.	Intron 2	'T' at the 82nd base of intron 2	'T'	T → G
6.		'C' at the 206th base of intron 2	C → G	'C'
7.		'A' at the 272nd base of intron 2	A → G	'A'
8.		'T' at the 338th base of intron 2	T → A	T → A
9.		'G' at the 1100th base of intron 2	G → A	G → A
10.		'C' at the 1247th base of intron 2	C → T	C → A
11.	Exon 3	'A' at the 138th base of exon 3	A → G	A → G

### *In silico* Characterization of Mutations in *OsGA20ox2* and *OsFBX267*

**Figure 4:**
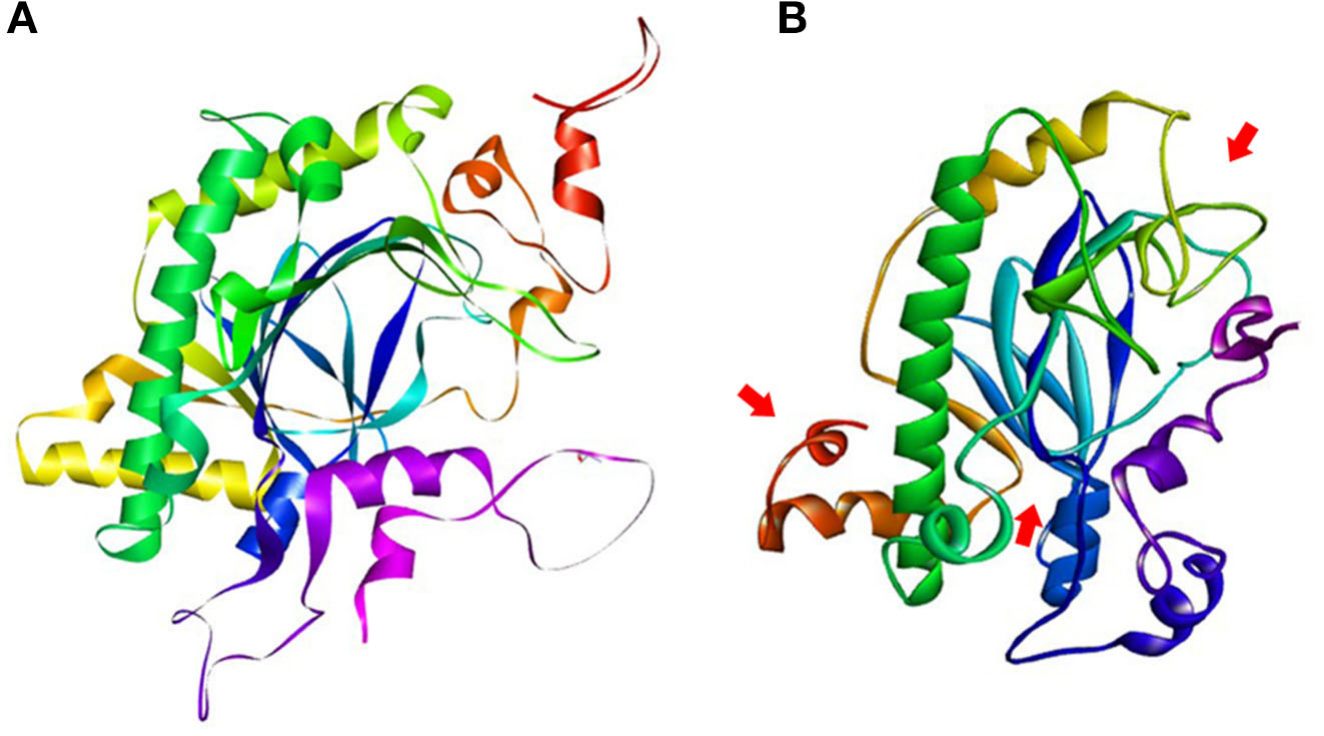
Protein structure prediction for the *OsGA20Ox2* gene. **(A)** IWP; **(B)** WP-22-2-loss of amino acids and structural changes is clearly visible in the mutant. The arrows indicate the visible structural changes and amino acid composition in the mutant WP-22-2.

**Figure 5:**
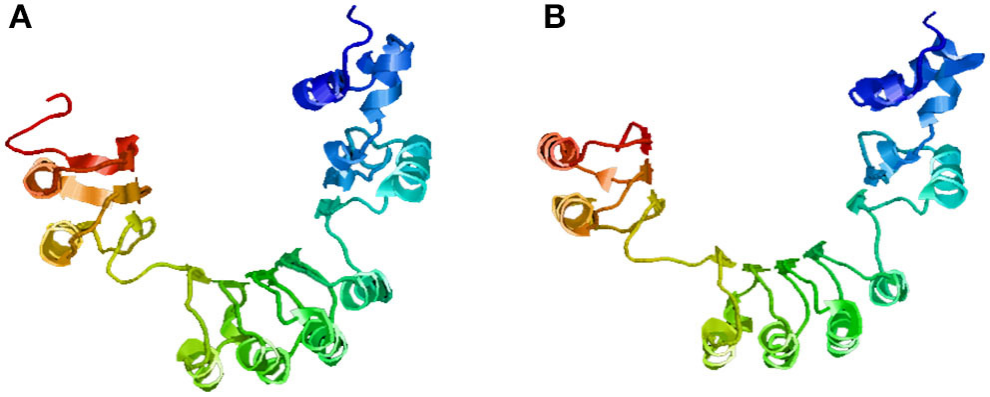
Protein structure prediction for the *OsFBX267* gene. **(A)** IWP; **(B)** WP-22-2—premature terminations and single nucleotide variations have caused structural changes in the mutant.

The SNPs and deletions in *OsGA20ox2* and *OsFBX267* were further analyzed. The *OsGA20ox2* gene of WP-22-2 had a deletion of 97 amino acids. This deletion results in a stop codon that truncates the protein, and, therefore, the protein expressed by *OsGA20ox2* gene would be non-functional. The structural differences in the protein expressed by *OsGA20ox2* of WP-22-2 and IWP are shown in Figure 4. In the case of *OsFBX267*, a comparison of sequence data showed 20 nucleotide substitutions. Among these substitutions, T → C and C → T transitions were prominent (six and five times, respectively, from IWP to WP-22-2) (Figure 5, Table 2). Of these 20 SNPs, 7 and 13 were synonymous and non-synonymous (12 missense and 1 non-sense) substitutions, respectively. Noteworthy, a G to A transition at 44th base in WP-22-2 converted a tryptophan codon to a stop codon, resulting in premature termination of translation. The effect of base changes in *OsGA20ox2* and *OsFBX267* were validated using SIFT (Ng and Henikoff, 2003) and PROVEAN (Choi et al., 2012; Choi and Chan, 2015) scores. The SIFT scores indicated that four amino acid substitutions affected the protein function, while the PROVEAN predicted that three amino acid substitutions were deleterious (Table 4). However, these scores have minimal relevance and correlation with the protein functions, as both *OsGA20ox2* and *OsFBX267* of WP-22-2 undergo a premature termination. Thus, while either of the genes does not produce functional proteins, this study suggests the implication of these genes in regulating early maturity and semi-dwarfism in WP-22-2 (Figures 4, 5). Thus, the loss of function of *OsGA20ox2* and *OsFBX267* has evidently promoted early maturity and semi-dwarfism in WP-22-2.

**Table 4:** Sorting Intolerant From Tolerant (SIFT) and PROVEAN scores for mutations in the OsFBX267 gene of WP-22-2 and its predicted effects on the protein.

**S. No**.	**Base changes (IWP, [base position], WP-22-2)**	**Amino acid changes (IWP, [a.a. position], WP-22-2)**	**Type of mutation**	**SIFT**		**PROVEAN**	
				**SIFT score**	**Predicted effect**	**Provean score**	**Predicted effect**
1.	T[36]C	Gly[12]Gly	Silent mutation	N/A	N/A	-	-
2.	C[41]T	Ser[14]Phe	Missense mutation	0.00	Affect protein function	−4.8	Deleterious
3.	G[44]A	Trp[15]STOP	Non-sense mutation	Functional protein (N/A)	N/A	−19.719	Deleterious
4.	T[97]C	Ser[33]Pro	Missense mutation	0.30	Tolerated	1.358	Neutral
5.	G[104]C	Arg[35]Pro	Missense mutation	0.03	Affect protein function	2.697	Neutral
6.	C[115]T	Pro[39]Ser	Missense mutation	0.15	Tolerated	−2.764	Deleterious
7.	T[129]C	Asp[43]Asp	Silent mutation	N/A	N/A	-	-
8.	C[156]T	Leu[52]Leu	Silent mutation	N/A	N/A	-	-
9.	A[163]G	Ile[55]Val	Missense mutation	0	Affect protein function	−0.292	Neutral
10.	A[175]C	Ile[59]Leu	Missense mutation	1	Tolerated	1.317	Neutral
11.	C[183]A	Ala[61]Ala	Silent mutation	N/A	N/A	-	-
12.	C[262]G & C[264]T	Leu[88]Val	Missense mutation	0.27	Tolerated	1.989	Neutral
13.	C[276]T	Arg[92]Arg	Silent mutation	N/A	N/A	-	-
14.	C[317]A	Ala[106]Glu	Missense mutation	0.22	Tolerated	−2.061	Neutral
15.	G[501]A	Prol[167]Pro	Silent mutation	N/A	N/A	-	-
16.	C[539]T	Leu[180]Pro	Missense mutation	0.06	Tolerated	−0.008	Neutral
17.	T[565]C	Cys[189]Arg	Missense mutation	0.75	Tolerated	2.089	Neutral
18.	T[588]C	Cys[196]Cys	Silent mutation	N/A	N/A	-	-
19	C[1087]G	His[363]Asp	Missense mutation	0	Affect protein function	1.806	Neutral

### Editing and Analysis of *OsGA20ox2* (*SD1*) in Pusa Basmati 1

**Figure 6:**
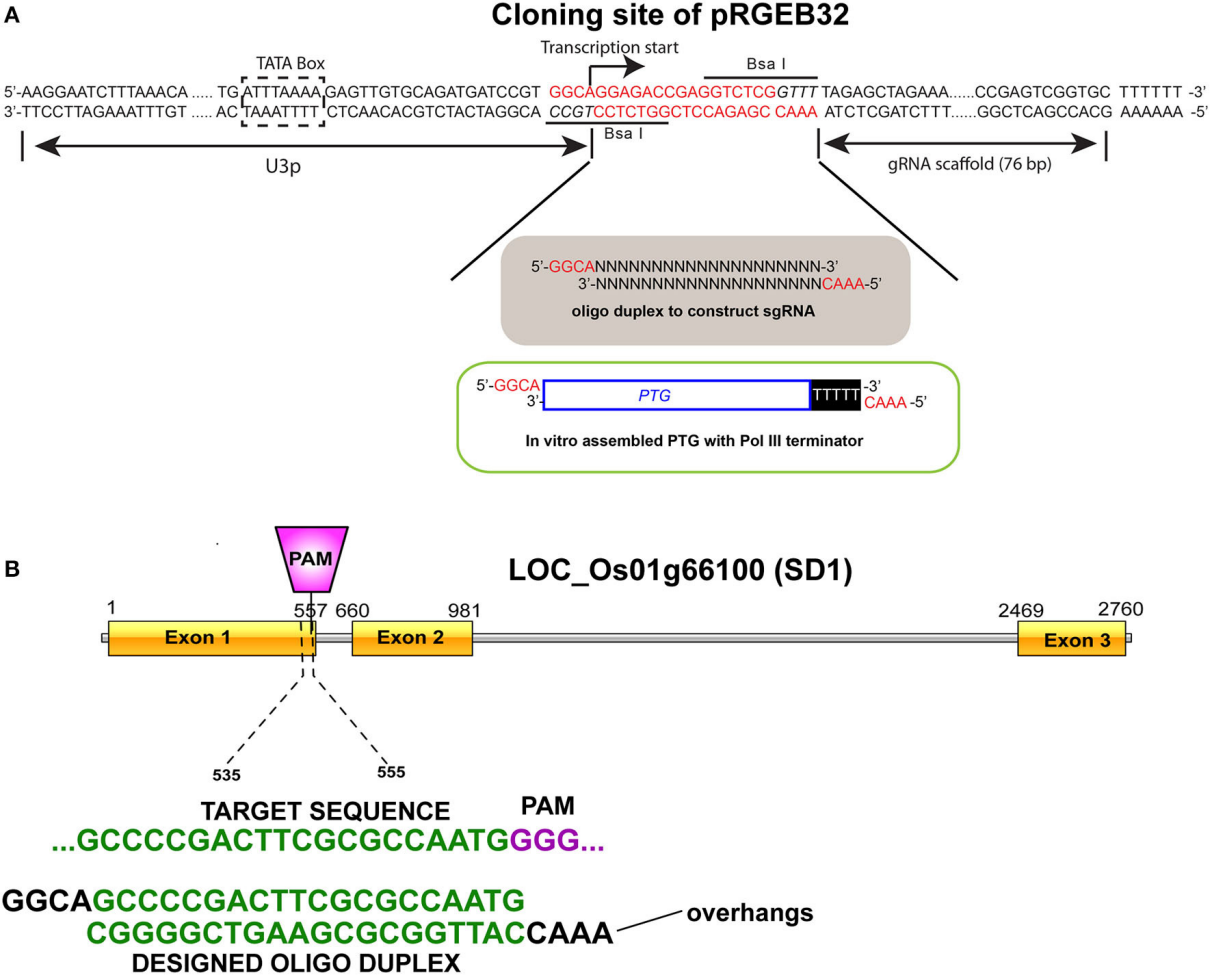
Single guard ribonucleic acid (sgRNA) for targeted genome engineering. **(A)** Cloning site of pRGEB32 plasmid when digested with *BsaI* restriction enzyme leaves a four-base overhang on either side. **(B)** Target site for sgRNA binding at the exon 1 of the *OsGA20ox2* gene (LOC_Os01g66100). A 20-nt oligonucleotide complex was designed and ligated with the pRGEB32 plasmid.

**Figure 7:**
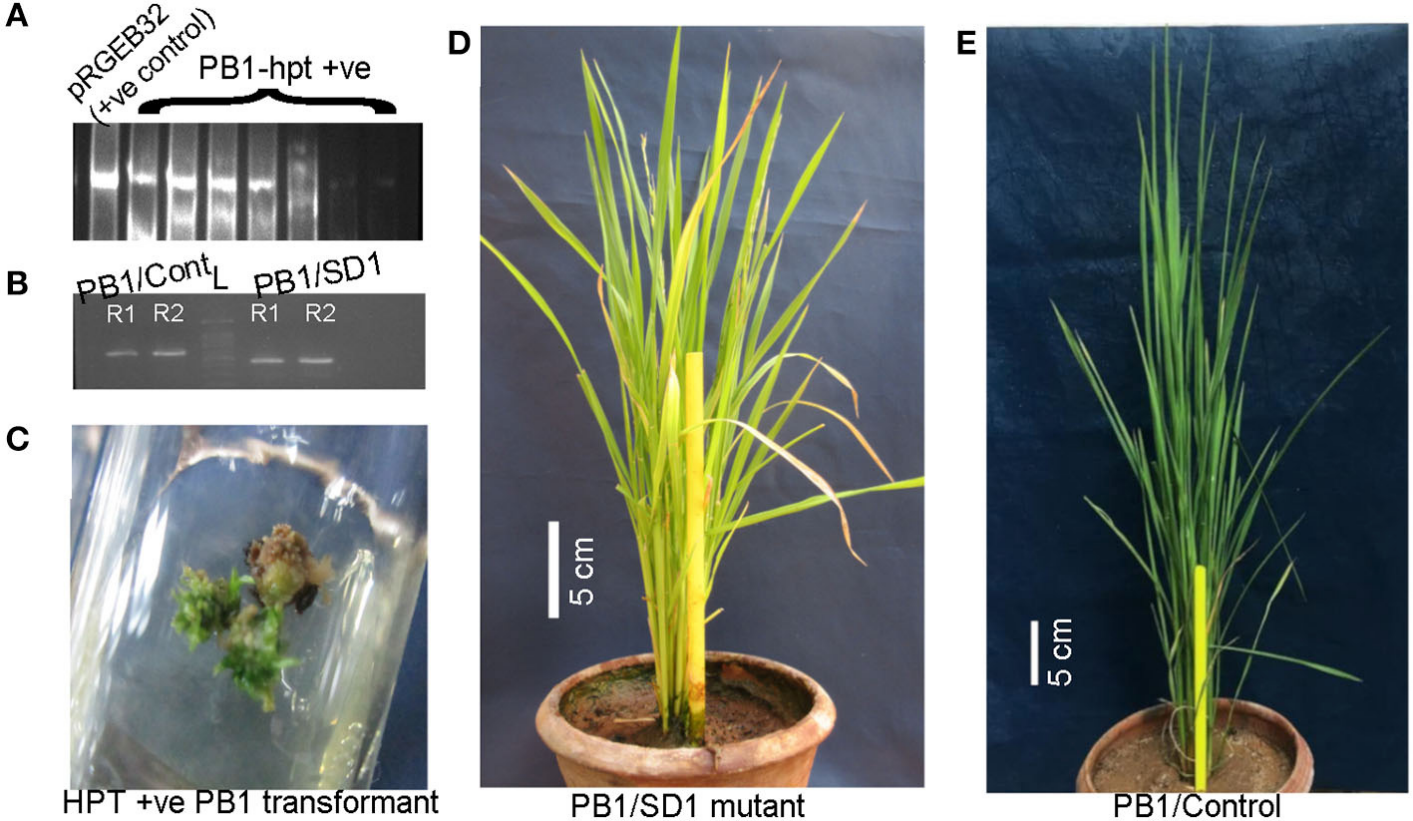
*Sd1* mutants of cultivar Pusa Basmati-1 (PB-1) were developed using CRISPR-Cas9. **(A)** HPTII screening of PB-1/SD1 transformants (lane 1—pRGEB32 plasmid as positive control, other lanes—PB-1/hpt positive transformants); (full-length gel presented in Supplementary Material 11); **(B)** A PB-1/SD1 mutant was observed with a small deletion in the exon 1 of the *OsGA20ox2* gene; lanes 1 and 2: biological duplicate of PB-1/Control and lanes 3 and 4: biological duplicate of PB-1/SD1 (cropped gel image; full-length gel is presented in Supplementary Material 12). **(C)** Shoot initiation from the hygromycin positive calli. **(D,E)** PB-1/SD1-mutant and PB-1-control respectively; scale bars: 5 cm.

Rice cultivar Pusa Basmati-1 offers a highly responsive system for tissue culture compared with other *indica-*type cultivars. To validate the mutation in *OsGA20ox2* identified in WP-22-2, the same gene in PB-1 was subjected to targeted genome-editing using the CRISPR-Cas9 approach (Figure 6). Around 100 seeds were inoculated for callus, and 80 healthy calli were co-cultivated with an *Agrobacterium* harboring CRISPR-Cas9-SD1 construct. The transformation produced seven hygromycin-positive (*hptII*) plants (Figure 7) with a transformation efficiency of 8.75%. Targeted amplification of *SD1* in T_0_ showed a deletion of several nucleotides, and that the plants were comparatively shorter (48 cm) compared with the control (PB-1; 95 cm). These preliminary data confirm the involvement of *SD1* in regulating plant height in rice; however, further functional characterization is necessary to delineate the precise mechanism underlying semi-dwarfism in *SD1* loss-of-function mutants.

## Discussion

This study deployed the use of γ-irradiation to achieve genomic changes in IWP, a popularly grown rice variety in Southern India. In addition, this study shows that the frequency of semi-dwarf mutants was higher in lower doses of γ- irradiation (100 and 200 Gy) than higher doses. The higher doses of mutagens cause lethality, which reduces the survival of the mutants (Shadakshari et al., 2001; Singh et al., 2006; Nayudu et al., 2007; Anilkumar, 2008). In addition, most of the morphological mutants identified in M_2_ generation failed to inherit in M_3_ generation. These characters may be controlled by recessive genes or are susceptible to the environment (Luo et al., 2012). The considerable shift in the means and variances between the M_2_ and M_3_ generations suggests the effects of recombination events in the mutants (Johnston, 2001; Siddiqui and Singh, 2010). The high heritability and high genetic advance observed in the M_6_ generation of mutants indicated that the traits were fixed in the selected genotypes or that undesirable traits were screened out. Based on the overall morphological superiority, high yield, fine grain structure, and similar cooking quality traits, the WP-22-2 mutant of IWP was selected as the superior mutant (Figure 1, Table 1).

Intercalary meristem cell division and elongation are the major causes for internodal elongation in rice, and deficiencies in these processes severely affect plant height. The earlier studies proved that the seedlings of WP-22-2 are highly sensitive to external GA_3_ application and this resulted in increase in seedling height similar to IWP. The gibberellin-sensitive mutants are primarily defective in genes controlling the gibberellin pathway of rice (Hedden and Sponsel, 2015). Semi-dwarfism in WP-22-2 is caused by the reduced elongation of internodes at the early stages of plant growth. In matured WP-22-2 plants, reduction in all four internodes was observed, resulting in sturdy plant architecture. After introducing the rice variety IR8, the OsGA20ox2 has been used as the semi-dwarfing gene for rice breeding throughout the world, and its role has been well studied (Tomita and Ishii, 2018). In plants, the GA20 oxidase converts GA intermediates into bioactive forms such as GA_1_ and GA_4_ (Yamaguchi, 2008); hence, loss of function may cause dwarfism in rice plants. The *OsGA20ox2* gene was downregulated in both IWP and WP-22-2. Genome sequence analyses show the presence of a 356-bp deletion in *OsGA20ox2*. The results confirm similar deletion mutations that have been reported earlier (Monna et al., 2002; Sasaki et al., 2002; Spielmeyer et al., 2002). It is evident from the findings that the deficiency caused loss of function and reduced the expression of the *OsGA20ox2* gene in WP-22-2. Furthermore, the observed differences in the expression of the *BRD2* gene (Supplementary Material 4) between the IWP and WP-22-2 emphasize the deficient gibberellin metabolism in the mutant causing semi-dwarfism. Significant changes in expression levels of more than one locus suggest an epistatic interaction of genes.

The deletion and SNPs observed (Monna et al., 2002; Spielmeyer et al., 2002) suggest the γ-rays induced mutability of the *OsGA20ox2* exon 1 region. In addition, four SNPs observed in intron 2 and exon 3 of the gene were unique to IWP and WP-22-2. The mutations had no visible lethal effects on other phenotypic traits and were found to be similar to the other semi-dwarf rice cultivars developed with the *SD1* gene. The presence of a functional *SD1* wild-type allele in tall plants and in few rice cultivars and landraces has been validated. A similar allele in semi-dwarf landraces indicates that *GA20ox2* is the vulnerable locus that causes semi-dwarfism in rice (Han et al., 2019). It offers key information that silencing this gene through targeted dmutagenesis, especially by CRISPR/Cas9, could create semi-dwarf mutants in a short time without causing lethal effects. To state further, the allelic variations of this region can be analyzed from diverse germplasm sets to effectively use this finding to identify mutable regions and to explore existing variations. This method could reduce the time required by conventional mutation breeding to select the best mutant lines without lethal effects.

Genome-wide searches to identify the gene(s) responsible for the early flowering in WP-22-2 were attempted with the NGS data. An F-box-containing protein, which is a potential target for early flowering, was identified in WP-22-2. F-box-containing proteins regulate photomorphogenesis, circadian clock and flowering time control in plants, and gibberellic acid signaling (Dill et al., 2004). Although the functions of only a few F-box-containing proteins were elucidated, many F-box-containing proteins are known to control flowering time and to cause gibberellin-insensitive dwarfs in rice (Sasaki et al., 2003). Multiple SNPs were identified in the *OsFBX267* (LOC_Os08g09460) gene of WP-22-2 from this investigation. Previous reports on the localized upregulation of this gene in floral parts similar to many heading date-related rice genes emphasize its role in earliness in flowering (Jain et al., 2007; Mohapatra et al., 2014). The multiple SNPs observed in the coding regions of *OsFBX267* resulted in a premature termination codon in exon 1 (Figure 5). The amino acid substitutions predicted were found to be highly deleterious to the normal function of this gene product. This information is the key finding in explaining the early maturity of WP-22-2 (Supplementary Material 9). However, the *in-silico* based analyses performed are preliminary, and further investigations with mapping populations could strengthen the prediction reported in this study.

As a proof of concept, we silenced the *OsGA20ox2* gene in the rice cultivar Pusa Basmati-1, a highly suitable variety for *Agrobacterium*-mediated transformation (Mohanty et al., 1999). The *Agrobacterium*-mediated transformation in PB-1 resulted in many hygromycin-positive plants (Figure 7). In one mutant, an allelic variation was observed in genome-edited PB-1 (CrSD1) than in control PB-1. This revealed the deletion affected by the CRISPR-Cas9-SD1 construct. In addition, the T_0_ plant was dwarf (48 cm) compared with the control (90–110 cm) and showed normal panicle exertion (Figure 7). The morphological observations and the PCR analyses of the genomic region showed that the CRISPR editing was successful in PB-1. However, a further analysis of the genome-wide effects of CRISPR on non-specific targets is essential.

## Conclusions

This study suggests the role of two genes, viz., *OsFBX267* and *OsGA20ox2*, in negatively influencing the height and maturity of rice, wherein mutations in these genes result in semi-dwarf and early flowering phenotypes. As M_6_ stable lines have been established, this could serve as a potential donor for developing new varieties for functional characterization and field trials. Mutation breeding has been a versatile tool being used for crop improvement. The advent of next-generation genomics has enabled the precise localization of mutations, which could be further exploited for targeted improvement of yield-contributing agronomic and climate-resilient traits. This study is an excellent example of coupling classical mutagenesis with NGS to identify two candidate genes, which were further studied. Also, targeted editing of *OsGA20ox2* in Pusa Basmati1 has been demonstrated to validate the findings. Altogether, the study has founded a base for further research on *OsFBX267* and *OsGA20ox2* to investigate their roles in regulating dwarfism and early flowering in this crop. This also opens the avenue for exploring these genes in other related grass species through comparative genome mapping approaches.

## Data Availability Statement

The datasets presented in this study can be found in online repositories. The names of the repository/repositories and accession number(s) can be found in the article/Supplementary Material.

## Author Contributions

MP conceptualized and designed the study. MA-P-L and RS performed the experiments. MA-P-L, RS, KK, JY, MM, and MP analyzed and interpreted the results. MA-P-L wrote the initial draft of the manuscript. JY and MM edited and revised the manuscript. All the authors read and approved the manuscript.

## Funding

This research was supported by the Vice-Chancellor's Fellowship for Excellence in Research awarded to MA-P-L, by the Tamil Nadu Agricultural University, Coimbatore, Tamil Nadu, India. Grant No: Dean (SPGS)/Vice-Chancellor's Fellowship/M.T.A./GPB/2017.

## Conflict of Interest

The authors declare that the research was conducted in the absence of any commercial or financial relationships that could be construed as a potential conflict of interest.

## Publisher's Note

All claims expressed in this article are solely those of the authors and do not necessarily represent those of their affiliated organizations, or those of the publisher, the editors and the reviewers. Any product that may be evaluated in this article, or claim that may be made by its manufacturer, is not guaranteed or endorsed by the publisher.
