# Supplementary material for: Loss of Function of OsFBX267 and OsGA20ox2 in Rice Promotes Early Maturing and Semi-Dwarfism in γ-Irradiated IWP and Genome-Edited Pusa Basmati-1
Source: Front Plant Sci. 2021 Sep 22;12:714066. doi: 10.3389/fpls.2021.714066 (PMC8494130; doi:10.3389/fpls.2021.714066)
Supplement: Supplementary file 1 [file Data_Sheet_1.PDF]

Supplementary Material

Supplementary material S1. Mean, variability and heritability estimates of morphological traits in M_2 generation of Improved White Ponni (IWP) generated by Gamma irradiation

**Treatments**	**Days to 50% flowering**		**Plant height (cm)**	
	Mean	Variance	Mean	Variance
Wildtype	111.50 ± 0.41		162.39 ± 1.18	
100 Gy	106.23 ± 1.44	62.60	153.43 ± 1.77	94.40
200 Gy	107.93 ± 1.48	49.84	144.04 ± 2.29	157.39
300 Gy	109.17 ± 1.20	42.97	138.68 ± 2.16	139.60
400 Gy	112.87 ± 0.54	8.81	141.96 ± 1.61	78.00
	Number of Productive Tillers		Panicle length (cm)	
Wildtype	17.23 ± 0.62		24.25 ± 0.14	
100 Gy	18.62 ± 0.73	15.77	23.27 ± 0.23	1.59
200 Gy	20.53 ± 0.79	18.83	23.73 ± 0.28	2.29
300 Gy	20.70 ± 0.68	13.80	24.86 ± 0.24	1.77
400 Gy	25.27 ± 1.18	41.51	25.08 ± 0.24	1.77
	Number of grains per panicle		Primary culm length (cm)	
Wildtype	196.07 ± 1.94		138.14 ± 1.20	
100 Gy	197.43 ± 5.18	804.87	130.16 ± 1.75	91.37
200 Gy	190.20 ± 3.98	475.82	120.41 ± 2.25	151.77
300 Gy	182.87 ± 3.01	271.98	113.82 ± 2.22	147.84
400 Gy	179.47 ± 3.42	349.91	116.88 ± 1.66	82.40
	Secondary culm length (cm)		1000 grain weight (g)	
Wildtype	127.52 ± 0.64		16.79 ± 0.03	
100 Gy	121.37 ± 1.44	32.16	16.64 ± 0.06	0.12
200 Gy	113.52 ± 1.99	119.04	16.54 ± 0.09	0.24
300 Gy	108.52 ± 2.46	181.50	16.52 ± 0.09	0.22
400 Gy	110.99 ± 1.55	71.91	16.64 ± 0.09	0.23
	Single plant yield (g)			
Wildtype	34.59 ± 1.38			
100 Gy	47.10 ± 1.83	100.60		
200 Gy	51.11 ± 2.21	147.05		
300 Gy	41.38 ± 1.64	80.24		
400 Gy	45.15 ± 3.28	322.36		

The values are mean ± standard error; the experiment was conducted as a completely randomized block design

Supplementary material S2. Mean, variability and heritability estimates of morphological traits in M3 generation of Improved White Ponni generated by gamma irradiation

Treatments[a]	Mean	PV	GV	PCV	GCV	H2	GA%	CD	Mean	PV	GV	PCV	GCV	H2	GA %	CD
	Number of grains per panicle								Primary culm length (cm)							
Wildtype	174.23								116.54							
100 Gy	178.50^{NS}	930.13	789.90	14.80	13.64	84.92	25.90	24.12	88.77**	272.33	268.80	18.59	18.47	98.70	37.80	3.83
200 Gy	180.63**	417.00	321.49	11.30	9.92	77.10	17.95	20.08	91.92**	203.60	197.96	15.52	15.30	91.23	30.09	4.88
300 Gy	191.15**	506.52	409.24	11.77	10.58	80.79	19.60	19.64	101.52**	81.06	77.78	8.87	8.70	95.94	17.52	3.61
400 Gy	169.70**	706.43	588.25	13.68	12.50	83.27	23.48	23.31	98.78**	34.30	30.40	5.93	5.58	88.59	10.82	4.24
	Secondary culm length (cm)								1[st]Internodal length (cm)							
Wildtype	105.48								43.56							
100 Gy	83.93**	252.75	250.52	18.94	18.85	99.12	38.67	3.04	41.52^{NS}	27.53	25.37	16.23	15.58	92.15	30.82	3.00
200 Gy	88.19**	183.49	180.00	15.36	15.21	98.11	31.04	3.82	30.33**	48.78	46.44	23.02	22.47	95.28	45.19	3.11
300 Gy	97.80**	82.89	79.97	9.30	9.14	96.48	18.50	3.40	35.45**	12.38	10.94	9.92	9.32	88.37	18.06	2.39
400 Gy	95.14**	30.56	28.02	5.81	5.56	91.70	10.98	3.41	35.09**	7.28	5.39	7.70	6.61	74.00	11.72	2.95
	2[nd]Internodal length (cm)								3[rd]Internodal length (cm)							
Wildtype	27.43								18.44							
100 Gy	26.27^{NS}	33.83	31.52	26.76	25.83	93.19	51.36	3.09	11.57**	17.64	16.13	32.14	30.73	91.48	60.56	2.50
200 Gy	20.21**	23.36	24.25	25.52	24.47	92.00	48.35	2.98	11.83**	15.17	13.60	32.91	31.16	89.62	60.76	2.60
300 Gy	24.84**	10.44	8.42	13.00	11.72	81.25	21.76	2.78	16.44**	9.14	6.71	18.38	15.75	73.50	27.82	3.10
400 Gy	24.07**	7.00	4.36	11.00	8.67	62.24	14.10	3.49	16.57**	3.08	0.84	10.60	5.54	27.36	5.97	3.21
	4[th]Internodal length (cm)								1000 grain weight (cm)							
Wildtype	8.41								16.32							
100 Gy	7.89**	5.02	4.45	33.51	31.56	88.68	61.23	1.53	16.32^{NS}	0.05	0.04	1.41	1.30	83.56	2.44	0.19
200 Gy	5.77**	4.11	3.60	35.15	32.87	87.48	63.34	1.47	16.50^{NS}	0.06	0.04	1.48	1.28	75.08	2.30	0.25
300 Gy	8.11^{NS}	3.96	2.77	24.51	20.54	70.21	35.45	2.16	16.43^{NS}	0.06	0.05	1.53	1.35	77.63	2.45	0.24
400 Gy	7.41**	2.23	0.60	20.40	10.40	25.92	10.91	2.80	16.70**	0.02	0.01	0.78	0.56	50.81	2.40	0.20

PV- phenotypic variance; GV-genotypic variance; PCV-phenotypic coefficient of variation; GCV-genotypic coefficient of variation; GA%-genetic advance as per cent of mean; H^2-heritability; CD-critical difference (5% level of significance); **-significant at 1% ($P<0.01$); NS-non-significant.The experiments were conducted in a randomized block design with 3 replications each;

frontiers

Supplementary material S3. Mutants exhibiting altered morphological traits observed in M2 and M3 generation, following gamma ray treatment of Improved White Ponni rice cultivar

**Traits**	**Classification Based on phenotype**	**No. of mutant progenies identified**	**Days to flowering**	
			**IWP (wild type)**	**Mutants**
Flowering	Early	5	112	79 – 92**
	Late	3	112	118-122**
Plant height	Tall			-
	Dwarf	11	162 cm	85-105 cm**
Tillering habit	High	4	18	32 – 44**
	Low	-	-	-
Narrow rolled leaf mutants		14	Normal	Narrow rolled leaves
Upper albino leaf mutant		1	Normal	Upper leaf albino
Lodging		5	Susceptible	Resistant
Grassy and extreme dwarf mutant		2	Normal	Grassy tillers
Lanky culm mutants		2	Normal	Thin culms(Lanky)

**-significant at 1% level of significance (P<0.01)

Supplementary material S4. Mean internode and second leaf lengths of IWP, WP-22-2 (control) and WP-22-2 (GA_3 treated)

	**Trait**	**Mean (cm)**	**Student's t-test with**	**t-stat**
IWP (wild-type)	1st internode	6.96 ± 0.29	WP-22-2 (untreated)	5.21^{**}
			WP-22-2 (GA3)	0.48^{ns}
	2nd leaf	11.20 ± 0.54	WP-22-2 (untreated)	1.33^{ns}
			WP-22-2 (GA3)	-3.28^{**}
	Seedling height	18.16 ± 0.34	WP-22-2 (untreated)	4.61^{**}
			WP-22-2 (GA3)	-3.35^{**}
WP-22-2 (untreated)	1st internode	5.36 ± 0.09	IWP	5.21^{**}
			WP-22-2 (GA3)	-3.32^{**}
	2nd leaf	10.26 ± 0.46	IWP	1.33^{ns}
			WP-22-2 (GA3)	-4.26^{**}
	Seedling height	15.62 ± 0.43	IWP	4.61**
			WP-22-2 (GA3)	-5.62**
WP-22-2 (GA3)	1st internode	6.72 ± 0.41	IWP	0.48^{ns}
			WP-22-2 (untreated)	-3.23^{**}
	2nd leaf	14.90 ± 0.99	IWP	-3.28^{**}
			WP-22-2 (untreated)	-4.26^{**}
	Seedling height	21.62 ± 0.97	IWP	-3.35^{**}
			WP-22-2 (untreated)	-5.62^{**}

Supplementary material S5. Log C_T values of the qRT-PCR for the six genes of IWP and WP-22-2 mutant

**Time**	**IWP**	**WP-22-2**	**Time**	**IWP**	**WP-22-2**
	SLR1		KO2		
0h	1.00 ± 0.44	1.00 ± 0.36	0h	1.00 ± 0.14	1.00 ± 0.09
6h	1.21 ± 0.32	0.67 ± 0.15	6h	4.46± 0.92	0.76 ± 0.09
12h	0.54 ± 0.04	1.29 ± 0.13	12h	1.64 ± 0.07	1.23 ± 0.10
24h	0.62 ± 0.11	1.12 ± 0.05	24h	0.65 ± 0.18	0.60 ± 0.04
	GA		MAX2		
0h	1.00 ± 0.21	1.00 ± 0.19	0h	1.00 ± 0.39	1.00 ± 0.15
6h	0.58 ± 0.89	0.25 ± 0.35	6h	1.73 ± 0.23	0.90 ± 0.33
12h	0.43 ± 0.18	0.56 ± 0.54	12h	1.29 ± 0.31	1.25 ± 0.16
24h	0.46 ± 0.16	0.42 ± 0.05	24h	1.51 ± 0.29	2.04 ± 0.15
	OsKOL4		BRD2		
0h	1.00 ± 0.70	1.00 ± 0.32	0h	1.00 ± 0.22	1.00 ± 0.13
6h	0.62 ± 0.35	0.69 ± 0.10	6h	1.07± 0.34	0.56 ± 0.03
12h	0.45 ± 0.28	0.78 ± 0.26	12h	2.00 ± 0.10	0.94 ± 0.14
24h	0.27 ± 0.46	0.63 ± 0.04	24h	2.04 ± 0.14	0.49 ± 0.08

Supplementary material S6. Raw reads of quantitative real time PCR and calculated double delta cycle threshold (ddCt) vlues for Improved White Ponni

Sample	Detector	Ct1	Ct2	Ct3	sd	Avg Ct	End CTl	dCt	Control 0h	ddCt	Log Ct	SE
0h	ACT	18.69043	18.75143	18.87199	0.092395	18.77128	18.77128	0				0.053346
6h		19.71252	18.9366	18.68244	0.536613	19.11052	19.11052	0	0	0	1	0.309823
12h		17.52496	17.55961	17.85235	0.179851	17.64564	17.64564	0	0	0	1	0.10384
24h		17.9862	17.54359	17.74342	0.221654	17.75774	17.75774	0	0	0	1	0.127975
0h	SLR1	20.28587	20.32007	18.96864	0.770566	19.8582	18.77128	1.086913				0.444899
6h		20.46152	19.36186	19.95824	0.550484	19.9272	19.11052	0.816683	1.086913	-0.27023	1.206001	0.317831
12h		19.64917	19.54738	19.70339	0.079203	19.63331	17.64564	1.987673	1.086913	0.900759	0.535605	0.045729
24h		19.48393	19.37917	19.74424	0.187978	19.53578	17.75774	1.778041	1.086913	0.691128	0.619369	0.108532
0h	GA	25.6159	24.92342	25.50591	0.372138	25.34841	18.77128	6.577128				0.21486
6h		25.66852	28.24972	25.47259	1.549917	26.46361	19.11052	7.353089	6.577128	0.775961	0.583999	0.894871
12h		25.78413	25.27166	25.24306	0.304467	25.43295	17.64564	7.787306	6.577128	1.210178	0.432215	0.175789
24h		25.32564	25.25902	25.76962	0.277571	25.45143	17.75774	7.693691	6.577128	1.116563	0.461191	0.16026
0h	KOL4	18.91882	16.78746	18.87388	1.217773	18.19339	18.77128	-0.5779				0.703102
6h		19.8539	18.62809	19.21232	0.613129	19.23144	19.11052	0.120918	-0.5779	0.698815	0.616078	0.354001
12h		17.68692	18.61817	18.37659	0.483259	18.22723	17.64564	0.581589	-0.5779	1.159486	0.447672	0.279018
24h		18.26182	19.12209	19.87052	0.804999	19.08481	17.75774	1.327071	-0.5779	1.904969	0.267022	0.46478
0h	KO2	23.8713	23.40062	23.55816	0.239589	23.61003	18.77128	4.838745				0.138331
6h		22.7051	19.9472	22.72311	1.597501	21.7918	19.11052	2.681279	4.838745	-2.15747	4.461306	0.922345

12h		21.64135	21.77498	21.90005	0.129375	21.77213	17.64564	4.126486	4.838745	-0.71226	1.638368	0.074697
24h		22.94799	23.55428	23.14867	0.308864	23.21698	17.75774	5.459244	4.838745	0.620499	0.650446	0.178328
0h	MAX2	27.79202	28.9507	27.79071	0.669343	28.17781	18.77128	9.406525				0.386457
6h		27.27432	28.02622	27.8793	0.398525	27.72661	19.11052	8.616088	9.406525	-0.79044	1.729598	0.230095
12h		26.98088	26.06241	27.02566	0.54367	26.68965	17.64564	9.04401	9.406525	-0.36251	1.285665	0.313897
24h		26.89267	25.98406	26.83632	0.509098	26.57102	17.75774	8.81328	9.406525	-0.59325	1.508637	0.293937
0h	BRD2	24.8134	24.24503	24.95882	0.3772	24.67242	18.77128	5.901132				0.217783
6h		24.70153	25.59393	24.50011	0.582148	24.93185	19.11052	5.821332	5.901132	-0.0798	1.056872	0.336113
12h		22.35735	22.64313	22.6464	0.165948	22.54896	17.64564	4.903322	5.901132	-0.99781	1.996966	0.095813
24h		22.38419	22.86231	22.65498	0.239762	22.63383	17.75774	4.87609	5.901132	-1.02504	2.035018	0.138431

Supplementary material S7. Raw reads of quantitative real time PCR and calculated double delta cycle threshold (ddCt) values for WP-22-2

Sample	Detector	Ct1	Ct2	Ct3	sd	Avg Ct	End CTl	dCt	Control 0h	ddCt	Log Ct	SE
0h	ACT	17.91066	16.95991	17.59314	0.484031	17.4879	17.4879	0				0.279463
6h		17.94863	17.91927	17.92895	0.014962	17.93228	17.93228	0	0	0	1	0.008639
12h		18.09094	17.92024	17.83795	0.12904	17.94971	17.94971	0	0	0	1	0.074504
24h		17.80471	17.93565	17.86262	0.065617	17.86766	17.86766	0	0	0	1	0.037885
0h	SLR1	19.73574	18.51923	19.38003	0.62549	19.21167	17.4879	1.723763				0.361138
6h		19.94728	20.47171	20.26819	0.264394	20.22906	17.93228	2.296774	1.723763	0.573011	0.672212	0.152652
12h		19.44218	19.05212	19.43329	0.22268	19.3092	17.94971	1.359488	1.723763	-0.36428	1.287235	0.128568
24h		19.36375	19.38665	19.51731	0.082842	19.42257	17.86766	1.554912	1.723763	-0.16885	1.124163	0.04783
0h	GA	24.0503	24.08337	23.50576	0.324354	23.87981	17.4879	6.391905				0.187271
6h		25.98886	27.03075	25.96276	0.609207	26.32746	17.93228	8.395172	6.391905	2.003268	0.249434	0.351736
12h		25.5455	24.11123	25.84618	0.927145	25.16764	17.94971	7.217927	6.391905	0.826022	0.564082	0.535303
24h		25.54767	25.58244	25.42339	0.083615	25.51783	17.86766	7.650173	6.391905	1.258268	0.418045	0.048277
0h	KOL4	18.18572	18.49626	19.25698	0.551167	18.64632	17.4879	1.158414				0.318226
6h		19.49516	19.82666	19.54671	0.178381	19.62284	17.93228	1.69056	1.158414	0.532146	0.691525	0.102991
12h		19.01537	19.45115	19.91258	0.448667	19.4597	17.94971	1.509993	1.158414	0.351579	0.783726	0.259045
24h		19.75073	19.60051	19.70951	0.077615	19.68692	17.86766	1.819259	1.158414	0.660845	0.632508	0.044813
0h	KO2	22.31022	22.55098	22.27216	0.151192	22.37779	17.4879	4.889883				0.087294
6h		23.35701	23.2627	23.05149	0.156443	23.22373	17.93228	5.291451	4.889883	0.401568	0.757035	0.090325
12h		22.48925	22.72593	22.39104	0.172152	22.53541	17.94971	4.585695	4.889883	-0.30419	1.234723	0.099395
24h		23.45705	23.56999	23.42243	0.077165	23.48316	17.86766	5.615497	4.889883	0.725614	0.60474	0.044552
0h	MAX2	27.26208	27.70776	27.23951	0.264071	27.40312	17.4879	9.915216				0.152466
6h		27.69297	27.63817	28.66928	0.580142	28.00014	17.93228	10.06786	9.915216	0.152641	0.899602	0.334955
12h		27.45098	27.85261	27.32611	0.275109	27.54323	17.94971	9.593524	9.915216	-0.32169	1.249796	0.158839
24h		26.73261	26.50486	27.02924	0.262941	26.75557	17.86766	8.88791	9.915216	-1.02731	2.038216	0.151814
0h	BRD2	22.83349	23.17317	23.26857	0.22868	23.09174	17.4879	5.60384				0.132032
6h		24.39101	24.39998	24.29639	0.057391	24.36246	17.93228	6.430178	5.60384	0.826339	0.563959	0.033136

12h		23.64141	23.89803	23.40212	0.248006	23.64719	17.94971	5.697477	5.60384	0.093637	0.937157	0.14319
24h		24.36719	24.50148	24.64976	0.141341	24.50614	17.86766	6.638485	5.60384	1.034645	0.488136	0.081605

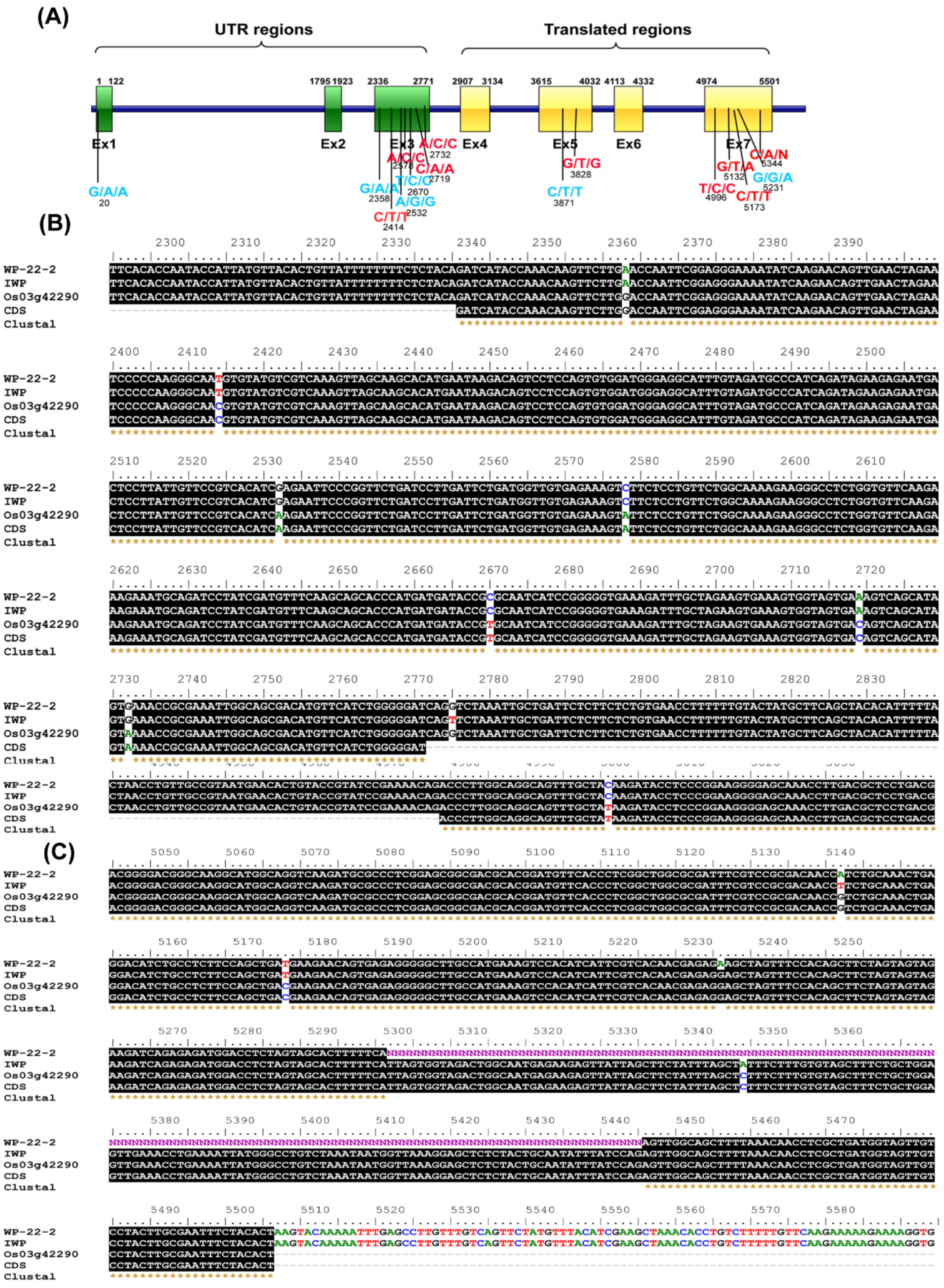

Supplementary material S8. Single nucleotide variations observed in the B3 DNA binding domain locus of WP-22-2. A) Gene illustration shows the location of SNPs in the gene. B&C)

Sequence alignments of exon 3 and exon 7 respectively. (Order of the gene sequences: WP-22-2, IWP, Annotated Nipponbare sequence LOC_Os03g42290 and coding sequences of the gene LOC_Os03g42290)

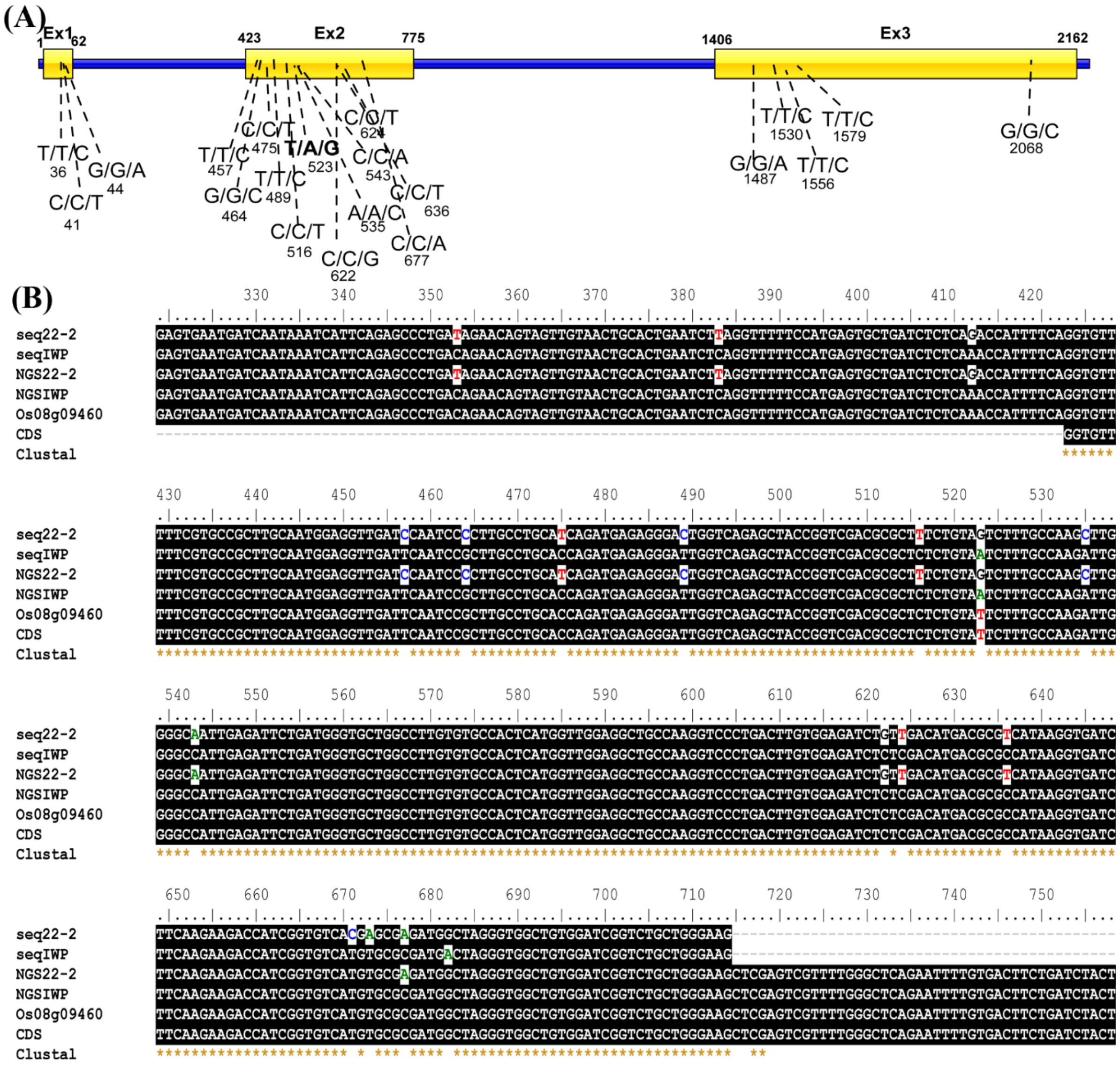

Supplementary material S9. Single nucleotide variations observed in the *OsFBX267* gene locus of WP-22-2. A) Gene illustration shows the location of SNPs observed between the Nipponbare/IWP/WP-22-2. B) Sequence alignments of exon 2 of *OsFBX267*. The sequences are aligned in order WP-22-2 (Sanger), IWP (Sanger), WP-22-2 (NGS), IWP (NGS), annotated gene sequence LOC_Os08g09460 and coding sequence of the gene

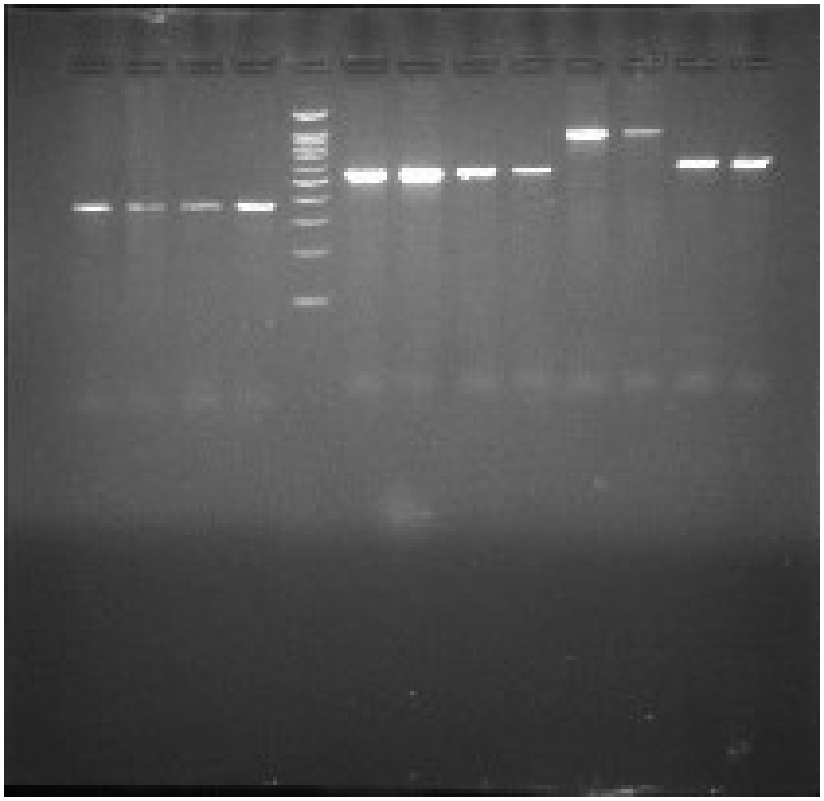

Supplementary material S10. Gel image of B3 DNA, FBX267 and GA20Ox2 gene regions of IWP and WP-22-2 (biological duplicates) amplified with PCR primers. IWP: 1&2, 5&6, 9&10; WP-22-2: 3&4, 7&8, 11&12; M: 100 bp ladder – each band represents a 100 bp increment from the lower band; A large deletion is observed between IWP and WP-22-2 in GA20Ox gene

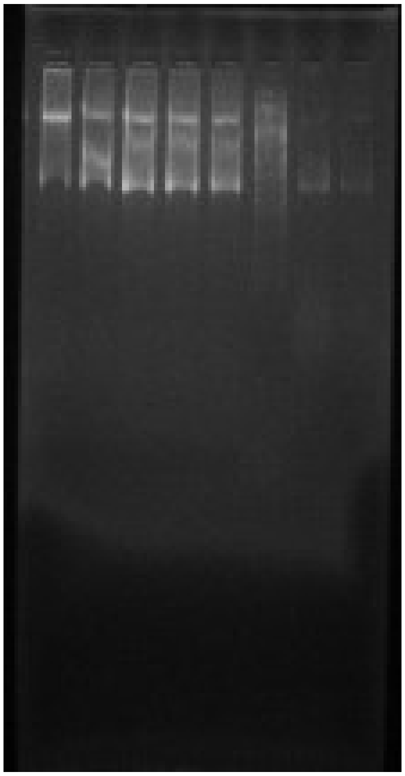

Supplementary material S11. HPTII screening of PB-1/SD1 transformants (lane 1 – pRGEB32 plasmid as positive control, other lanes – PB-1/hpt positive transformants);

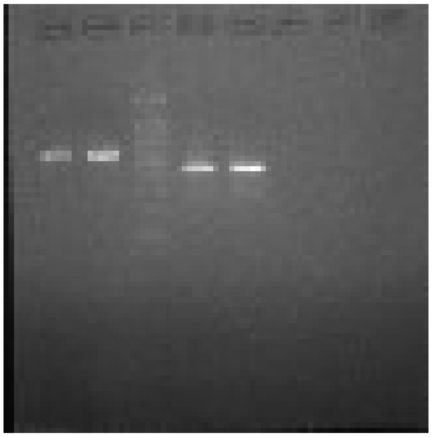

Supplementary material S12. A PB-1/SD1 mutant was observed with a small deletion in the exon 1 of *OsGA20ox2* gene; Lanes 1&2: biological duplicate of PB-1/Control and lanes 3&4: biological duplicate of PB-1/SD1.

Supplementary material S13.The designed sgRNA sequences for GA20Oxidase gene target and the gene specific primers used for characterization

**Primer ID**	**Sequence (5' to 3')**	**GC%**
Designed sgRNA		
CrsgRNA-SD1-F	GGCAGCCCCGACTTCGCGCCAATG	70.80
CrsgRNA-SD1-R	AAACCATTGGCGCGAAGTCGGGGC	62.50
Gene specific primers		
SD1_F	TCCCTCATCCCCTGTGGTG	55.00
SD1_R	ATGGCGGGTAGTAGTTGCAC	60.00
